# Partial Angular Sparse Representation Based DOA Estimation Using Sparse Separate Nested Acoustic Vector Sensor Array

**DOI:** 10.3390/s18124465

**Published:** 2018-12-17

**Authors:** Jianfeng Li, Zheng Li, Xiaofei Zhang

**Affiliations:** 1 College of Electronic and Information Engineering, Nanjing University of Aeronautics and Astronautics, Nanjing 211106, China; lizhengjsnj@163.com (Z.L.); zhangxiaofei@nuaa.edu.cn (X.Z.); 2 Key Laboratory of Dynamic Cognitive System of Electromagnetic Spectrum Space (Nanjing University of Aeronautics and Astronautics), Ministry of Industry and Information Technology, Nanjing 211106, China; 3 College of Computer and Information, Hohai University, Nanjing 211100, China

**Keywords:** partial angular sparse representation, DOA estimation, sparse separate nested acoustic vector sensor array, off-grid sources

## Abstract

In this paper, the issue of direction of arrival (DOA) estimation is discussed, and a partial angular sparse representation (SR)-based method using a sparse separate nested acoustic vector sensor (SSN-AVS) array is developed. Traditional AVS array is improved by separating the pressure sensor array and velocity sensor array into two different sparse array geometries with nested relationship. This improved array geometry can achieve large degrees of freedom (DOF) after the extended vectorization of the cross-covariance matrix, and only partial SR of the angle is required by exploiting the cyclic phase ambiguity caused by the large inter-element spacing of the virtual array. Joint sparse recovery is developed to amend the grid offset and unitary transformation is utilized to transform the complex atoms into real-valued ones. After sparse recovery, the sparse vector can simultaneously provide high-resolution but ambiguous angle estimation and unambiguous reference angle estimation embedded in the AVS array, and they are combined to obtain unique and high-resolution DOA estimation. Compared to other state-of-the-art DOA estimation methods using the AVS array, the proposed algorithm can provide better DOA estimation performance while requiring lower complexity. Multiple simulation results verify the effectiveness of the approach.

## 1. Introduction

Sensor arrays can utilize signals from multiple paths to overcome fading effect and enhance system capacity, so they have found wide application in many fields [1,2,3,4]. As a key issue for sensor array signal processing, direction of arrival (DOA) estimation has attracted lots of attention in many systems, e.g., radar [5,6], sonar [7], and wireless communication [8,9], to name a few. Compared to the conventional scalar sensor array, which only measures signal pressure information, the acoustic vector sensor (AVS) array provides a vector output, which contains both pressure and velocity information, so it can further extend array aperture and strengthen parameter identifiability [10]. Furthermore, the AVS array itself can provide unambiguous reference DOA estimation, which enables the inter-element spacing of the array to be larger than half-wavelength to enhance spatial resolution [11]. Since its introduction, DOA estimation using AVS array has attracted growing interest [11,12]. Many effective DOA estimation methods using AVS array have been developed, such as the estimation of signal parameters via rotational invariance technique (ESPRIT)-based method [11], Capon-based method [12], self-initiating multiple signal classification (MUSIC) method [13], propagator method (PM) [14], high-order decomposition-based method [15], and successive MUSIC-based methods [16,17]. However, the array geometries they used were simply evolutions of the conventional compact scalar array, which has limited degrees of freedom (DOF).

Recently, the research into sparse array geometries has been attractive, for they can achieve high DOFs in the co-array domain. Two well-known sparse array geometries are the coprime array [18] and the nested array [19]. The coprime array has large inter-element spacing and can achieve unique DOA estimation based on the coprime-ness [20,21], but its virtual array has many holes, which may enhance the sidelobe. The nested array can achieve *O*(*N*^2^) contiguous virtual elements in the co-array domain with only *O*(*N*) physical elements, and it has been applied in radar [22,23] and sonar [24] systems. In [25], the nested array concept was applied to AVS array signal processing, and the DOA could be estimated via tensor modelling, but only the AVS was adopted, instead of the pressure sensor, so the array geometry does not fit the AVS well. In [26], we separated the pressure sensor array and velocity sensor array in the AVS array into two nested geometries, generating high DOFs in the co-array domain, but sparse representation (SR) covering the whole angular range is required. Additionally, both the methods in [25,26] assume the sources are correctly located in the predefined grid, which actually cannot be guaranteed, no matter how fine the grid is [27]. The grid mismatch problem may intensively degrade the DOA estimation performance [28].

In this paper, we propose a sparse separate nested (SSN) AVS array, which separates the pressure sensor array and velocity sensor array into two different geometries, which have a nested relationship, while both have large inter-element spacing. This array geometry generates a long virtual array with large inter-element spacing in the co-array domain, and then only partial angular SR is required by exploiting the cyclic phase ambiguity caused by the large inter-element spacing. Meanwhile, unitary transformation is employed to transform the complex atoms into real-valued ones, and joint SR is formulated to amend the offsets caused by the off-grid sources. After sparse recovery, the non-zero positions and elements in the sparse vector can provide high-resolution but ambiguous angle estimation and unambiguous reference angle estimation, respectively. Finally, they are combined to obtain unique and high-resolution DOA estimation. Complexity analysis and multiple simulations verify that the proposed method outperforms the successive MUSIC [17], tensor-based [25] and SR-based methods [26] in terms of computation complexity, estimation accuracy, angle resolution and robustness to nonuniform noise.

The rest of the paper is organized as follows. Section 2 introduces the data model for the proposed SSN-AVS array. Section 3 shows the major steps of the proposed DOA estimation method, as well as some summaries and analyses. The simulation results are presented in Section 4 to verify the effectiveness of the proposed method, while the conclusions are made in Section 5.

Notation: ${\left(.\right)}^{T}$, ${\left(.\right)}^{*}$, ${\left(.\right)}^{H}$ and ${\left(.\right)}^{+}$ denote the transpose, conjugate, conjugate-transpose, and pseudo-inverse operations, respectively. E[.] means expectation. diag(***a***) means a diagonal matrix, with ***a*** being the diagonal. vec(.) is the vectorization of a matrix. ⊗ and ° denote the Kronecker product and Hadamard product, respectively. $I_{K}$ and ${\mathrm{II}}_{K}$ are *K* × *K* identity matrix and reverse identity matrix, respectively. ./ means element-wise division, and angle(.) means to extract the phase.

## 2. Data Model

**Figure 1:**
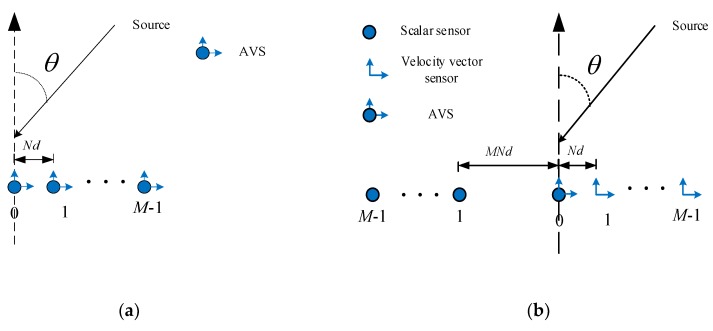
(**a**) Traditional AVS array; (**b**) the proposed SSN-AVS array.

A Traditional AVS geometry is shown in Figure 1a, where the *M* AVSs are uniformly arranged, with inter-element spacing being *Nd* (*d* is the unit spacing, which is generally set as half-wavelength). The AVS consists of a pressure scalar sensor and two identical but orthogonally oriented velocity vector sensors. Due to the directivity of the velocity sensors, the AVS array itself can provide unambiguous reference angle estimation [11,16], which enables the inter-element spacing to be larger than half-wavelength to enhance spatial resolution (the spacing is *Nd* in Figure 1a). However, as the pressure sensor array and the velocity sensor array have the same array geometry, which results in many redundant virtual elements in the difference co-array domain (we will discuss this in Section 3), the achieved DOF is limited. We propose an SSN-AVS array shown in Figure 1b, where the pressure sensors are extracted and arranged along the negative side with inter-element spacing being *MNd*. The pressure sensor and velocity sensors are no longer co-located but separated into two different geometries with nested relationship.

Assuming that there are *K* far-field uncorrelated source signals impinging on the proposed array, then the output of the pressure sensor array is formulated as

(1) $$x_{p}(t)=A_{p}s(t)+n_{p}(t)$$

where $s(t)={\left[s_{1}(t),s_{2}(t),\cdots ,s_{K}(t)\right]}^{T}$ is a vector containing all the *K* source signals, $n_{p}\left(t\right)$ is the additive white Gaussian noise vector, and $A_{p}=[a_{p}(\theta_{1}),a_{p}(\theta_{2}),\cdots ,a_{p}(\theta_{K})]$ denotes the direction matrix, which depends on the geometry of pressure sensor array. The steering vectors $a_{p}(\theta_{k}),k=1,\ldots ,K$ are expressed as

(2) $$a_{p}\left(\theta_{k}\right)=[1,e^{-j\pi MN\sin\theta_{k}},\cdots ,e^{-j\pi (M-1)MN\sin\theta_{k}}{]}^{T},k=1,\ldots ,K$$

Similarly, the outputs of the velocity vector sensor array are expressed as

(3) $$x_{v1}(t)=A_{v}\Phi_{1}s(t)+n_{v1}(t)$$

(4) $$x_{v2}(t)=A_{v}\Phi_{2}s(t)+n_{v2}(t)$$

where $\Phi_{1}=diag(\sin\theta_{1},\cdots ,\sin\theta_{K})$ and $\Phi_{2}=diag(\cos\theta_{1},\cdots ,\cos\theta_{K})$ are two diagonal matrices reflecting different components along different axes. $n_{v1}\left(t\right)$ and $n_{v2}(t)$ are additive white Gaussian noise vectors. $A_{v}=[a_{v}(\theta_{1}),a_{v}(\theta_{2}),\cdots ,$ $a_{v}(\theta_{K})]$ is the direction matrix of the velocity sensor array, and the steering vectors are expressed as

(5) $$a_{v}\left(\theta_{k}\right)=[1,e^{j\pi N\sin\theta_{k}},\cdots ,e^{j\pi (M-1)N\sin\theta_{k}}{]}^{T},k=1,\ldots ,K$$

According to Equation (2) and Equation (5), the steering vectors of the pressure sensor array and velocity sensor array satisfy nested relationship, which will greatly increase the DOF in the co-array domain shown in the next section. It is also noted that the proposed SSN-AVS array just separates the pressure array and velocity array, it does not add extra AVS, which can be observed from Figure 1, so the total AVS number is still *M*.

## 3. Partial Angular Sparse Representation Based DOA Estimation Method

### 3.1. Virtual Array in the Co-Array Domain

According to Equation (1) and Equation (3)–Equation (4), the cross-covariance matrix between $x_{v1}(t)$ and $x_{p}(t)$ is

(6) $$R_{vp1}=E[x_{v1}(t)x_{p}^{H}(t)]=A_{v}\Phi_{1}R_{s}A_{p}^{H}$$

where $R_{s}=E[s\left(t\right)s^{H}\left(t\right)]=$ $diag(\sigma_{1}^{2},\sigma_{2}^{2},\ldots ,\sigma_{K}^{2})$ is a diagonal matrix, whose diagonal elements are the signal powers $\sigma_{k}^{2},k=1,\ldots ,K$ [29]. The cross-covariance matrix used in Equation (6) can effectively reduce the influence of nonuniform noise.

Thereafter, to obtain the virtual array in the co-array domain, we vectorize the cross-covariance matrix as

(7) $$\begin{array}{ll} y_{vp1} & =vec(R_{vp1})=vec(A_{v}\Phi_{1}R_{s}A_{p}^{H})\\ & =(A_{p}^{*}\circ A_{v})p_{1}\\ & =A_{pv}p_{1} \end{array}$$

where $p_{1}=diag(\Phi_{1}R_{s})={\left[\sin\theta_{1}\sigma_{1}^{2},\ldots ,\sin\theta_{K}\sigma_{K}^{2}\right]}^{T}$ is a vector, and can be regarded as a new signal vector in the co-array domain. $A_{pv}=A_{p}^{*}\circ A_{v}\in C^{M^{2}\times K}$ can be regarded as a new direction matrix corresponding to a *M*^2^-element virtual array, whose steering vectors are expressed as

(8) $$a_{pv}(\theta_{k})=a_{p}^{*}(\theta_{k})⊗a_{v}(\theta_{k})={\left[1,e^{j\pi N\sin\theta_{k}},\cdots ,e^{j\pi (M^{2}-1)N\sin\theta_{k}}\right]}^{T},k=1,\ldots ,K$$

Based on Equation (2) and Equation (5), now an *M*^2^-element virtual array with inter-element spacing *Nd* can be achieved. Compared to the original physical array in Figure 1, the virtual array shows much larger DOF. Additionally, the new signal vector $p_{1}$ is a real-valued one, which means that the array aperture can be furtherly extended by combining the conjugate data of the vector in Equation (7). Construct the extended vector as

(9) $$\begin{array}{ll} y_{z1} & =\left[\begin{array}{c} {\mathrm{II}}_{M^{2}}y_{vp1}{}^{*}\\ y_{vp1}(1:M^{2}-1) \end{array}\right]=\left[\begin{array}{c} {\mathrm{II}}_{M^{2}}A_{pv}^{*}\\ A_{pv}(1:M^{2}-1,) \end{array}\right]p_{1}\\ & =Ap_{1} \end{array}$$

where $y_{vp1}(1:M^{2}-1)$ means to extract the first (*M*^2^−1) elements of $y_{vp1}$, and $A_{pv}(1:M^{2}-1,)$ means the first (*M*^2^ − 1) rows of $A_{pv}$. Here, we extract the first (*M*^2^ − 1) elements of $y_{vp1}$ to eliminate the overlapped origin element in the extended virtual array. $A={\left[A_{pv}^{H}{\mathrm{II}}_{M^{2}},A_{pv}{\left(1:M^{2}-1,\right)}^{T}\right]}^{T}$ is the extended direction matrix, whose steering vectors $a(\theta_{k}),k=1,\ldots ,K$ are expressed as

(10) $$a(\theta_{k})={\left[e^{-j\pi (M^{2}-1)N\sin\theta_{k}},e^{-j\pi (M^{2}-2)N\sin\theta_{k}},\ldots ,1,e^{j\pi N\sin\theta_{k}},\cdots ,e^{j\pi (M^{2}-1)N\sin\theta_{k}}\right]}^{T},k=1,\ldots ,K$$

Based on Equation (9), now a (2*M*^2^ − 1)-element virtual array with steering vectors being $a(\theta_{k})$ can be obtained, and the large DOF can be expected to improve the parameter estimation performance and system capacity. If the traditional array geometry in Figure 1a is used, then the virtual steering vector in Equation (8) will have many redundant elements due to the same geometry being used by the pressure array and velocity array. Additionally, the aperture extension in Equation (9) will be unavailable, and the final achievable DOF will be (2*M* − 1), which is much smaller than what the proposed geometry achieved.

It is shown that the steering vector in Equation (10) satisfies conjugate symmetrical property, so unitary transformation can be employed to transform the complex vector into real-valued one [30], which can effectively reduce the computation complexity.

The unitary matrix is defined as

(11) $$Q=\frac{1}{\sqrt{2}}\left[\begin{array}{ccc} I_{M^{2}-1} & 0 & jI_{M^{2}-1}\\ 0 & \sqrt{2} & 0\\ {\mathrm{II}}_{M^{2}-1} & 0 & -j{\mathrm{II}}_{M^{2}-1} \end{array}\right]$$

Then the extended steering vector can be transformed into a real-valued vector

(12) $$\begin{array}{l} a_{r}(\theta_{k})=Q^{H}a(\theta_{k})\\ =\sqrt{2}\times [\cos(\pi (M^{2}-1)N\sin\theta_{k}),\cdots ,\cos(N\pi \sin\theta_{k}),\\ 1/\sqrt{2},\sin(\pi (M^{2}-1)N\sin\theta_{k}),\cdots \sin(N\pi \sin\theta_{k}){]}^{T} \end{array}$$

According to Equation (9) and Equation (12), the real-valued data is obtained via

(13) $$\begin{array}{ll} y_{rz1} & =Q^{H}y_{z1}\\ & =Q^{H}Ap_{1}\\ & =A_{r}p_{1} \end{array}$$

where $A_{r}=[a_{r}(\theta_{1}),a_{r}(\theta_{2}),\cdots ,a_{r}(\theta_{K})]=Q^{H}A$ is the real-valued direction matrix.

Based on $x_{v1}(t)$, $x_{p}(t)$ and the steps from Equation (6) to Equation (13), now the real-valued data $y_{rz1}$ is obtained. Similarly, based on $x_{v2}(t)$ and $x_{p}(t)$, whose cross covariance matrix is $R_{vp2}=E[x_{v2}(t)x_{p}^{H}(t)]=A_{v}\Phi_{2}R_{s}A_{p}^{H}$, another real-valued vector $y_{rz2}$ can be obtained

(14) $$y_{rz2}=A_{r}p_{2}$$

where $p_{2}={\left[\cos\theta_{1}\sigma_{1}^{2},\ldots ,\cos\theta_{K}\sigma_{K}^{2}\right]}^{T}$. Combining $y_{rz1}$ and $y_{rz2}$ yields

(15) $$\begin{array}{ll} y_{z} & =y_{rz1}+jy_{rz2}=A_{r}(p_{1}+jp_{2})\\ & =A_{r}[e^{j\theta_{1}}\sigma_{1}^{2},\ldots ,e^{j\theta_{K}}\sigma_{K}^{2}]\\ & =A_{r}p \end{array}$$

where $p=[e^{j\theta_{1}}\sigma_{1}^{2},\ldots ,e^{j\theta_{K}}\sigma_{K}^{2}]$ contains unambiguous DOA information, which is embedded in the AVS array (generated from $\Phi_{1}$ and $\Phi_{2}$).

Now a virtual array with output being $y_{z}$ is obtained, and the direction matrix $A_{r}$ is real-valued, and sparse representation-based DOA estimation can be developed.

### 3.2. Partial Angular Sparse Representation

Suppose that the angle grid of interest is $\left\{{\tilde{\theta }}_{1},{\tilde{\theta }}_{2},\cdots ,{\tilde{\theta }}_{P}\}(P\gg K\right)$, which contains the true DOAs. Then construct the dictionary $\Omega =[a_{r}({\tilde{\theta }}_{1}),\ldots ,a_{r}({\tilde{\theta }}_{P})]$, which must contain the columns of $A_{r}$, so Equation (15) can be written as

(16) $$y_{z}=\Omega \rho$$

where ρ is a *P*-element vector, which only has *K* non-zero elements from p, and the positions of the non-zero elements give the DOA estimations. However, according to Equation (10), the inter-element spacing of the (2*M*^2^ − 1)-element virtual array is *Nd*, which means phase ambiguities in this array. Let $z_{p}=e^{jN\pi \sin{\tilde{\theta }}_{p}}$ denotes the phase difference between adjacent elements corresponding to angle ${\tilde{\theta }}_{p}$, then there are another (*N* − 1) angles ${\tilde{\theta }}_{p,n},n=2,\ldots ,N$ satisfying

(17) $$e^{jN\pi \sin{\tilde{\theta }}_{p,n}}=z_{p},n=2,\ldots ,N$$

**Figure 2:**
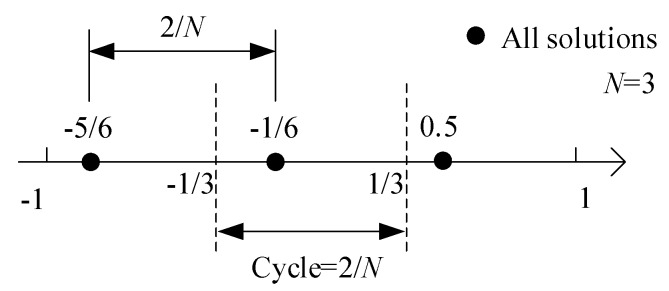
The cyclic solution distribution (*N* = 3).

The phase ambiguity shown in Equation (17) will cause multiple false angle estimations. However, we can in turn exploit the ambiguity to shrink the size of the dictionary and focus on one specific cycle. It is derived from Equation (17) that the *N* angles satisfy

(18) $$\sin{\tilde{\theta }}_{p,n}=\sin{\tilde{\theta }}_{p}-\frac{2n_{v}}{N},n=2,\ldots ,N$$

where $n_{v}$ is an integer ensuring $\sin{\tilde{\theta }}_{p,n}$ located in the range [−1, 1]. According to Equation (18), the *N* solutions ($\sin{\tilde{\theta }}_{p}$ and $\sin{\tilde{\theta }}_{p,n},n=2,\ldots ,N$) are uniformly distributed within the range [−1, 1] with adjacent distance being 2/*N*. Figure 2 shows an example when $\sin{\tilde{\theta }}_{p}=0.5$ and *N* = 3, and other two ambiguous solutions are $\sin{\tilde{\theta }}_{p,2}=-1/6$ and $\sin{\tilde{\theta }}_{p,3}=-5/6$, respectively. If we divide the range [−1, 1] into *N* cycles with each width being 2/*N*, then one cycle provides only one solution. Therefore, we can choose one specific cycle as a representative to construct the dictionary, and recover other solutions based on their uniform distribution after sparse vector recovery. Without loss of generality, we choose range [−1/*N*, 1/*N*], whose corresponding angular range and dictionary are [arcsin(−1/N),arcsin(1/N)] and $\Omega_{rep}$, respectively. Then the sparse representation can be expressed as

(19) $$y_{z}=\Omega_{rep}\rho$$

where the elements in $\rho \in {\mathbb{C}}^{P\times 1}$ corresponding to the representative solutions are the same as those in p, and the others are zero. For example, as Figure 2 shows, the true solution 0.5 is represented by −1/6 in $\Omega_{rep}$.

Now the sparse representation based on partial angular domain is conducted, and the phase ambiguity can be avoided. However, Equation (19) is established based on the assumption that the representative angles are right located in the pre-defined grid, but the angles are very likely to lie off the grid, no matter how fine the grid is defined. The grid mismatch will degrade the sparse recovery performance. Consequently, an improved sparse presentation framework should be developed to tackle the off-grid problem.

### 3.3. Joint Sparse Representation Framework

In this section, we take the off-grid angles into account, and utilize the joint sparse representation framework to enhance the robustness to grid mismatch.

Denote the new grid within the partial angular range [arcsin(−1/N),arcsin(1/N)] as ${\tilde{\theta }}_{1},{\tilde{\theta }}_{2},\cdots ,{\tilde{\theta }}_{Q}$, which is uniformly sampled. Then the true representative angle ${\bar{\theta }}_{k}$ can be represented by a nearest grid angle ${\tilde{\theta }}_{q,k}$ plus an offset $\alpha_{k}$(maybe zero when the true angle is right on the gird). Based on first-order Taylor expansion around the predefined grid, the true steering vector can be approximately expressed as [28,31]

(20) $$a({\bar{\theta }}_{k})\approx a({\tilde{\theta }}_{q,k})+\frac{\partial a({\tilde{\theta }}_{q,k})}{\partial {\tilde{\theta }}_{q,k}}\alpha_{k}$$

Then, Equation (19) has a robust form

(21) $$y_{z}=(\Omega_{rep}+\Omega_{rep}^{\prime }\Lambda )\rho$$

where $\Omega_{sub}^{\prime }=\left[\frac{\partial a({\tilde{\theta }}_{1})}{\partial {\tilde{\theta }}_{1}},\ldots ,\frac{\partial a({\tilde{\theta }}_{Q})}{\partial {\tilde{\theta }}_{Q}}\right]$ denotes the derivatives on the sampling grid, Λ=diag(β) is a diagonal matrix containing the offsets, and

(22) $$\beta (q)=\{\begin{array}{l} \alpha_{k},\;if\;{\tilde{\theta }}_{q,k}={\tilde{\theta }}_{q},k=1,\ldots ,K\\ 0,\;\;\;\;\;\;\;others \end{array},q=1,\ldots ,Q$$

So β is also a sparse vector with *K* non-zero elements. Let ω=Λρ, then ω and ρ are joint sparse. Equation (21) is rewritten as

(23) $$y_{z}=\Omega_{rep}^{\prime }\rho +\Omega_{rep}^{\prime }\omega$$

Equation (23) is a joint sparse recovery problem, and joint orthogonal matching pursuit (JOMP) [31] can be utilized to recover the sparse vectors **ρ** and **ω**. The basic steps of JOMP are shown in Table 1. It is noted that the grid can be coarse due to offset compensation afterwards [28], so the sparse recovery complexity can be reduced.

### 3.4. Ambiguity Elimination and DOA Estimation

After sparse recovery using JOMP, the estimations of ρ and ω can be obtained. The positions of non-zero elements in ρ give the grid angles ${\tilde{\theta }}_{q,k},k=1,\ldots ,K$, which are nearest to the representative angles. Meanwhile, the offsets are obtained via

(24) β=diag(ρ./ω)

From Equation (24), the offsets $\alpha_{k},k=1,\ldots ,K$ can be acquired, then the angles estimated in the representative range are

(25) $${\bar{\theta }}_{k}={\tilde{\theta }}_{q,k}+\alpha_{k},k=1,\ldots ,K$$

The angles obtained in Equation (25) are representative angles. Finally, we need to resolve the ambiguity problem in the angle estimation. According to Equation (18), from one specific angle ${\bar{\theta }}_{k}$, we can recover a total of *N* angles.

To determine the true angle in the *N* angles, we need an unambiguous reference angle estimation, which is actually embedded in the sparse vector ρ, whose non-zero elements give the estimation of $p=[e^{j\theta_{1}}\sigma_{1}^{2},\ldots ,e^{j\theta_{K}}\sigma_{K}^{2}]$. Then the unambiguous reference angle is obtained via

(26) $${\overset{⌣}{\theta }}_{k}=angle(p(k)),k=1,\ldots ,K$$

By finding the angle nearest to ${\overset{⌣}{\theta }}_{k}$ in the *N* angles, the true DOA estimation can be determined. It should be noted that the ambiguous angle and unambiguous reference angle are automatically paired due to the sparse vector.

### 3.5. Remarks and Summary

**Remark 1:** In practice, the cross-covariance matrix in Equation (6) is estimated via finite snapshots

(27) $$R_{vp1}=\frac{1}{T}\sum_{t=1}^{T}\left(x_{v1}(t)x_{p}^{H}(t\right))$$

where T denotes the number of snapshots.

**Remark 2:** One-dimensional angles are assumed in this paper, which can find its applications in some situations where the signals are in the same plane with the arrays, such as sea-surface detection, satellite-to-satellite location, ground signal finding and so on.

**Remark 3:** For DOA estimation methods, including the proposed method, the source number *K* is generally assumed to be known a priori. The source number estimation is another important issue for array signal processing [32].

The major steps of the proposed algorithm are:

1. Construct the cross-covariance matrix between the pressor sensor array and velocity sensor array via Equation (27);
2. Vectorize the cross-covariance matrix to obtain a vector and combine its conjugate to form a (2*M*^2^ − 1)-element virtual array with inter-element spacing being *Nd*;
3. Employ unitary transformation to transform the complex direction matrix into a real-valued one and form the final output via Equation (15);
4. Construct a dictionary covering the partial angular domain [arcsin(−1/N),arcsin(1/N)] and establish the joint sparse representation framework via Equation (23);
5. After sparse recovery based on JOMP, obtain the grid and offset estimations and combine them to obtain the representative angle estimation using Equation (25);
6. Recover all angle estimations based on the representative angle, and determine the final DOA estimation based on the unambiguous angle embedded in the sparse vector.

Based on the steps shown above, the proposed method requires neither EVD nor peak searching, and only requires a real-valued dictionary covering the partial angular domain, so the main complexity of the proposed method lies in cross-covariance matrix construction and joint sparse recovery. The theoretical complexity (measured by the number of complex multiplications) of the proposed method is shown in Table 2, where the main complexities of the Successive MUSIC [17], tensor-based method [25] and SR-based method [26] are also presented for comparison. The same AVS numbers are used, and *n*_1_ denotes the local search steps used by Successive MUSIC, *M*_1_ = *M*^2^/4 + *M*/2, *n*_2_ denotes the global search steps used by tensor-based method, *n*_3_ denotes the dictionary size of the SR-based method, *n*_4_ denotes the dictionary size of the proposed method. A typical setting is: *M* = 4, *T* = 200, *K* = 2, *n*_1_ = 60, *n*_2_ = 90, *n*_3_ = 90, *n*_4_ = 60. It is indicated in Table 2 that the proposed method costs less computation resources than other methods.

## 4. Simulation Results

In the simulations, the proposed SSN-AVS array is configured as *M* = 4 and *N* = 2, which means there are totally 4 AVSs. For the other methods, the successive MUSIC adopts traditional array shown in Figure 1a, the tensor-based method adopts nested AVS array and the SR-based method uses separated nested AVS array. The total AVS numbers used are the same for fair comparison. Assume there are two uncorrelated signals with DOAs being $\theta_{1}=20.76{}^{\circ}$ and $\theta_{2}=45.34{}^{\circ}$, respectively. *T* = 200 snapshots are collected, and the root mean square error (RMSE) of the angle estimation is defined below to measure the DOA estimation performance

(28) $$RMSE=\frac{1}{K}\sum_{k=1}^{K}\sqrt{\frac{1}{L}\sum_{l=1}^{L}\left[{\left({\hat{\theta }}_{k,l}-\theta_{k}\right)}^{2}\right]}$$

where ${\hat{\theta }}_{k,l}$ denotes the estimation of $\theta_{k}$ of the *l*-th Monte Carlo trial, whose total number is *L* = 500.

**Figure 3:**
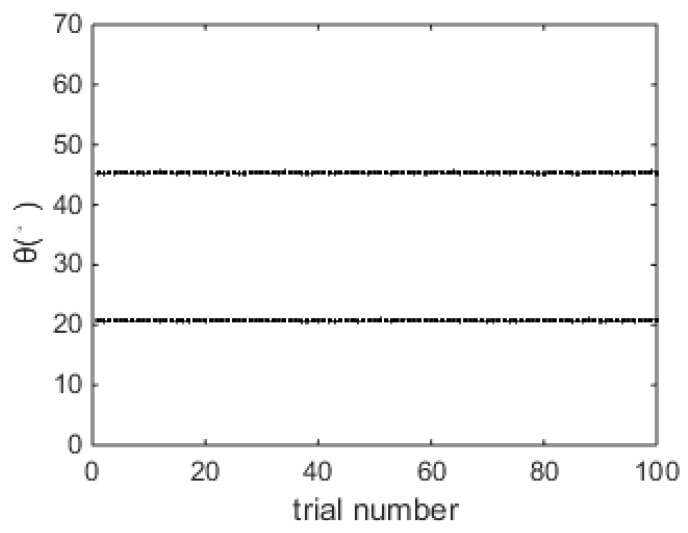
DOA estimation results over 100 trials (SNR = 0dB).

Figure 3 shows the DOA estimation results of the proposed algorithm over 100 trials when SNR = 0 dB, and it is shown that both the two DOAs can be accurately estimated.

**Figure 4:**
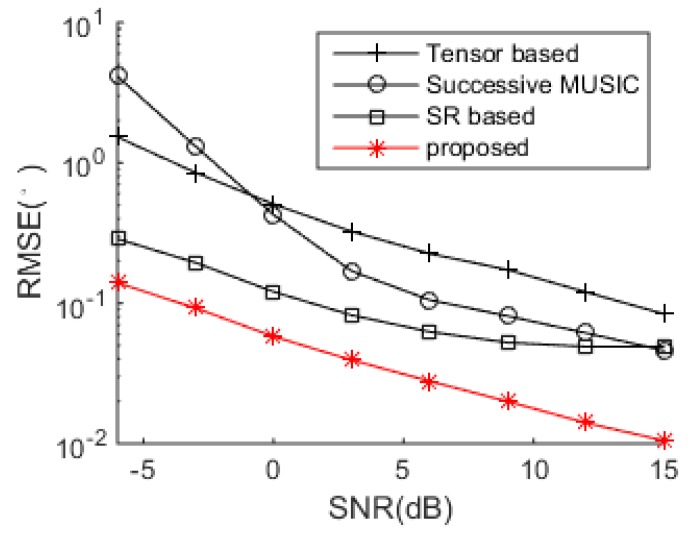
DOA estimation performance comparison.

**Figure 5:**
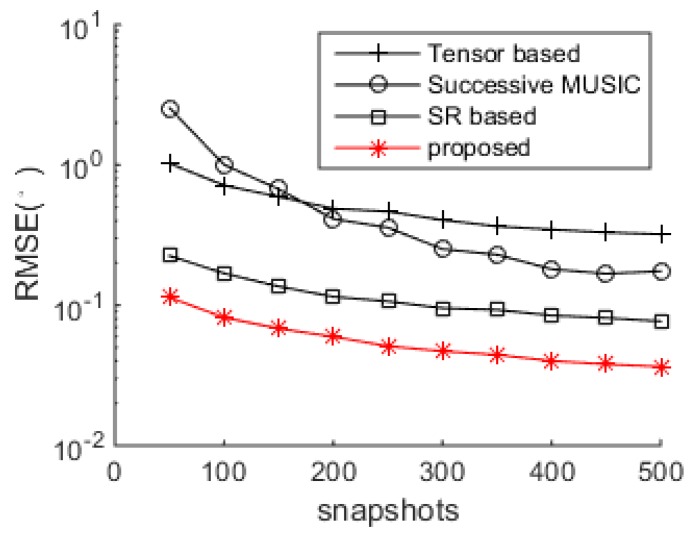
DOA estimation performance comparison versus snapshots (SNR = 0dB).

**Figure 6:**
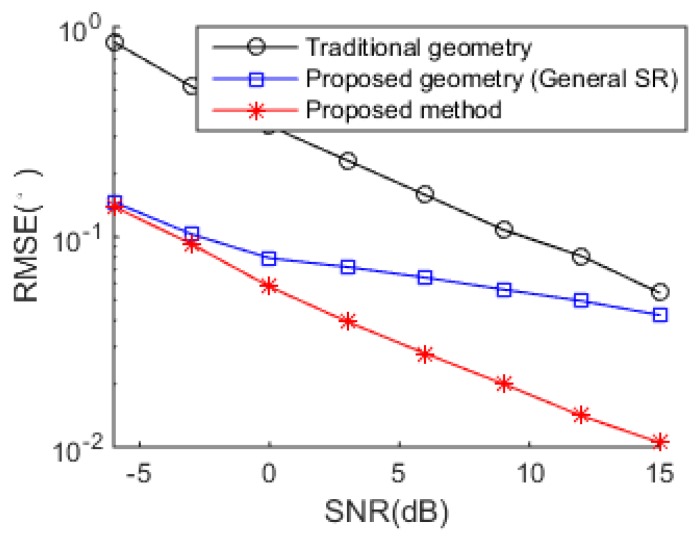
DOA estimation performance improvement contributor verification.

The DOA estimation performance comparisons between the successive MUSIC [17], tensor-based method [25], SR-based method [26] and the proposed algorithm versus SNR and snapshot number are shown in Figure 4 and Figure 5, respectively. The grid interval used by the successive MUSIC, tensor-based method and SR-based method is 0.1°, while the grid interval of the proposed method is 1°, which can save much complexity in the sparse recovery. It is shown in Figure 4 and Figure 5 that the proposed method achieves better DOA estimation performance than the other three methods. One reason is the large DOF and aperture generated by the proposed SSN-AVS array, and the other reason is that the proposed method can amend the grid offsets caused by the off-grid sources, which lead to the performance degradations of the other three methods, despite a finer grid they used. To verify the two reasons that lead to the performance improvement, we test the proposed method under other two configurations: 1. uses traditional array geometry, 2. uses the proposed geometry but utilizes general SR instead of joint SR in Section 3.3. The DOA estimation performance comparison between these three situations has been shown in Figure 6, which indicates that the both the array geometry and joint SR contribute to the performance improvement, especially the array geometry. As explained in Section 3.3, the aperture extension is unavailable and the final achievable DOF will be greatly reduced if the traditional array geometry is used.

**Figure 7:**
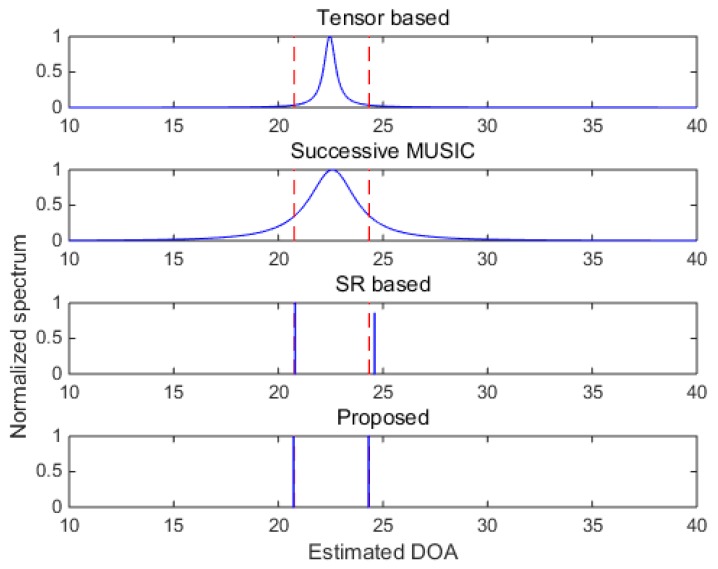
DOA estimation results with closely spaced sources (SNR = 5 dB).

**Figure 8:**
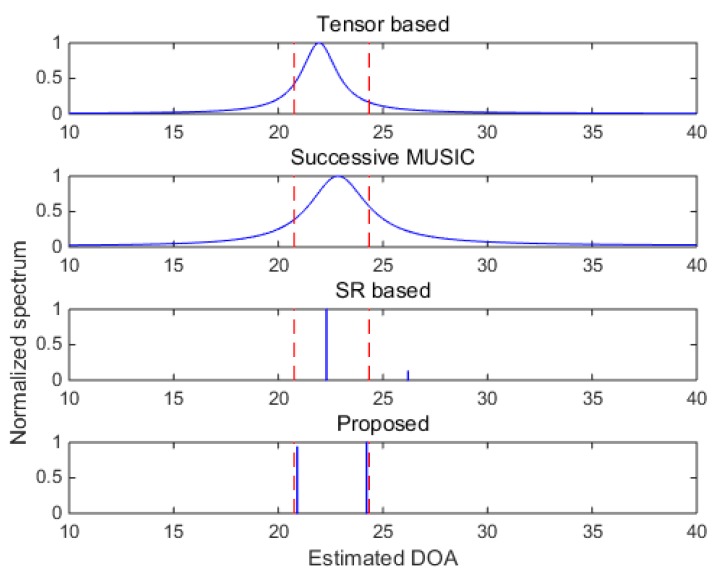
DOA estimation results with closely spaced sources (SNR = −5 dB).

**Figure 9:**
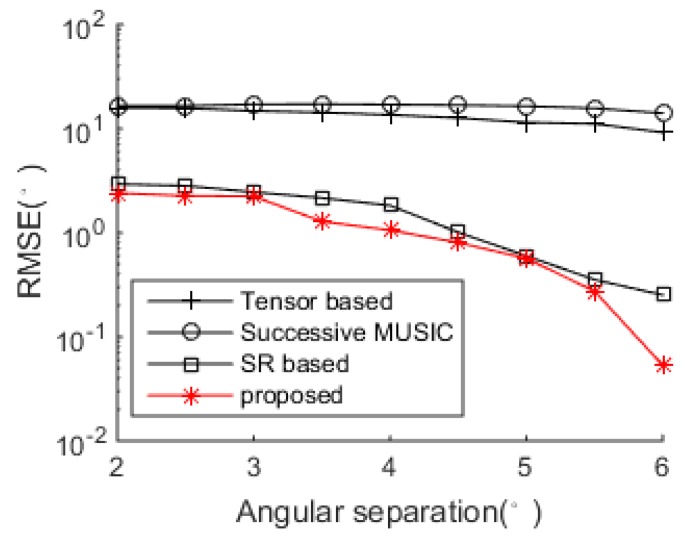
DOA estimation performance comparison versus angular separation (SNR = 5 dB).

In Figure 7 and Figure 8, two closely spaced sources with DOAs respectively being $\theta_{1}=20.76{}^{\circ}$ and $\theta_{2}=24.34{}^{\circ}.34{}^{\circ}$ are adopted to test the resolutions of the algorithms. When SNR = 5 dB, subspace-based methods including the tensor-based method and successive MUSIC fail to identify the two closely spaced sources, while the SR-based method and the proposed method still work well due to the super-resolution property of sparse recovery technique. When SNR gets lower in Figure 8, the SR-based method fails, and the proposed method can approximately identify the two sources (with visible deviations). Consequently, the proposed method shows finer angular resolution than the other methods. To further evaluate the resolution performances of the methods, Figure 9 shows the DOA estimation performance comparison versus angular separation when SNR = 5 dB. The reference angle is set as $\theta_{1}=40.36{}^{\circ}$, and the second angle is $\theta_{2}=\theta_{2}+∆\theta$, where ∆θ denotes the angular separation varying among the range [2°,6°]. It is shown that the subspace-based methods cannot achieve effective results within the given range, and the SR-based method and the proposed method work better, especially when ∆θ>4° (can clearly identify the two sources). Meanwhile, the proposed method always achieves the best DOA estimation performance versus the variation of the angular separation.

**Figure 10:**
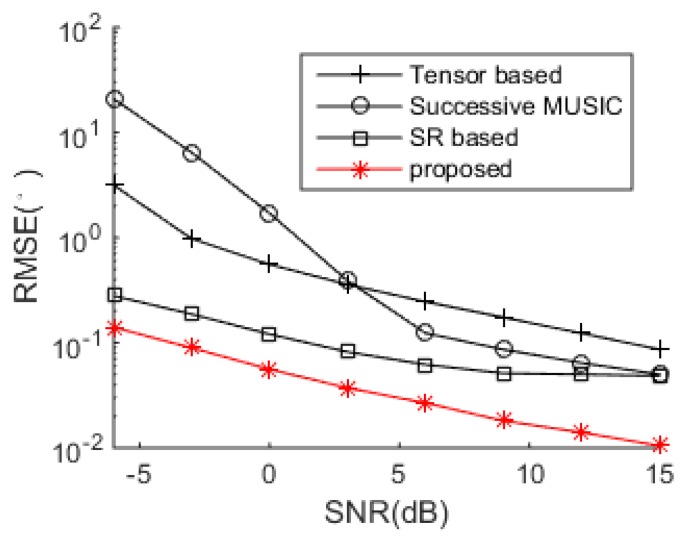
DOA estimation performance comparison with non-uniform spatial noise.

Figure 10 shows the DOA estimation performance comparison with non-uniform spatial noise. Compared to Figure 4, both the tensor-based method and successive MUSIC method have performance degradations. The subspace-based methods are sensitive to nonuniform noise due to the permeation between the signal and noise subspaces. Both the SR-based method and the proposed method is robust to nonuniform noise due to the usage of cross covariance matrix, which can effectively restrain the power of nonuniform noise.

## 5. Conclusions

In this paper, the SSN-AVS array is designed and a corresponding partial angular SR-based DOA estimation method is proposed. Multiple analyses and simulations verify that the proposed method has the following advantages:

1. It requires neither EVD nor peak searching, and only requires a real-valued dictionary covering the partial angular domain with a coarse grid, thereby having low complexity.
2. Based on the SSN-AVS array, it obtains large DOF and aperture, which can improve DOA estimation performance and spatial resolution.
3. It is robust to off-grid sources based on the joint SR formulation.
4. It is robust to non-uniform noise due to the usage of the cross-covariance matrix.

## Figures and Tables

**Table 1:** Basic steps for JOMP.

**Input:**$y_{z}$, $\Omega_{rep}$, $\Omega_{rep}^{\prime }$, *K*
**Initial**: $r=y_{z}$, $F_{1}=\emptyset ,F_{2}=\emptyset$
For *k* = 1: *K* *t* = $\underset{n}{\max}{\left[\Omega_{rep}{\left(:,n\right)}^{T}r\right]}^{2}+{\left[\Omega_{rep}^{\prime }{\left(:,n\right)}^{T}r\right]}^{2}$ $F=[F_{1}\cup \Omega_{rep}(:,t),F_{2}\cup \Omega_{rep}^{\prime }(:,t)]$ $q=F^{+}y_{z}$ $r=y_{z}-Fq$ end
$q={\left[q_{1}^{T},q_{2}^{T}\right]}^{T}$ ^1^
**output**: $\rho =q_{1}$, $\omega =q_{2}$

^1^q is halved to obtain $q_{1}$ and $q_{2}$.

**Table 2:** Complexity comparison.

Algorithms	Complex Multiplications	Typical Setting
Successive MUSIC	9*M*^2^*T* + 27*M*^3^ + 2*M*^2^*K* + 2*K*^3^ + 9*n*_1_*M*^2^*K*	47,888
Tensor-based	9*M*^2^*T* + 4*M*_1_^3^ + 45 *M*_1_^2^ + 9 *M*_1_^2^*K* + *n*_2_(9*M*_1_^2^ + 3*M*_1_)	62,712
SR-based	5*M*^2^*T* + 2*M*^4^ + *n*_3_*M*^2^ + O(2*M*^2^*n*_3_^2^)	536,352
Proposed	2*M*^2^*T* + *K*(4*n*_4_*M*^2^ − 2*n*_4_ + 10 *M*^2^)	14,160
